# A critique of the European Commission Document, “State of the Art Assessment of Endocrine Disrupters”

**DOI:** 10.3109/10408444.2012.690367

**Published:** 2012-05-26

**Authors:** Lorenz R. Rhomberg, Julie E. Goodman, Warren G. Foster, Christopher J. Borgert, Glen Van Der Kraak

**Affiliations:** 1 Gradient, Cambridge, MA, USA; 2 Department of Obstetrics and Gynecology, McMaster University, Hamilton, Ontario, Canada; 3 Applied Pharmacology and Toxicology, Inc., Gainesville, FL, USA; 4 Department of Physiological Sciences, University of Florida College of Veterinary Medicine, Gainesville, FL, USA; 5 Department of Integrative Biology, University ofGuelph, Guelph, Ontario, Canada

**Keywords:** Endocrine active, endocrine disruptor, WHO/IPCS, weight of evidence, state of science

## Abstract

In this commentary, we critique a recently finalized document titled “State of the Art Assessment of Endocrine Disrupters” (SOA Assessment). The SOA Assessment was commissioned by the European Union Directorate-General for the Environment to provide a basis for developing scientific criteria for identifying endocrine disruptors and reviewing and possibly revising the European Community Strategy on Endocrine Disrupters. In our view, the SOA Assessment takes an anecdotal approach rather than attempting a comprehensive assessment of the state of the art or synthesis of current knowledge.To do the latter, the document would have had to (i) distinguish between apparent associations of outcomes with exposure and the inference of an endocrine-disruption (ED) basis for those outcomes; (ii) constitute a complete and unbiased survey of new literature since 2002 (when the WHO/IPCS document, “Global Assessment of the State-of-the-Science of Endocrine Disruptors” was published); (iii) consider strengths and weaknesses and issues in interpretation of the cited literature; (iv) follow a weight-of-evidence methodology to evaluate evidence of ED; (v) document the evidence for its conclusions or the reasoning behind them; and (vi) present the evidence for or reasoning behind why conclusions that differ from those drawn in the 2002 WHO/IPCS document need to be changed. In its present form, the SOA Assessment fails to provide a balanced and critical assessment or synthesis of literature relevant to ED. We urge further evidence-based evaluations to develop the needed scientific basis to support future policy decisions.

## 1 Introduction

In response to recommendations from the Intergovernmental Forum on Chemical Safety in 1997, the World Health Organization/International Programme on Chemical Safety (WHO/IPCS) published a survey and evaluation in 2002 titled “Global Assessment of the State-of-the-Science of Endocrine Disrupters” (WHO, 2002). This document described a framework for evaluating information from diverse data sets in a structured manner to “provide an objective, global assessment of the current state-of-the-science relative to environmental endocrine disruption [ED] in humans, experimental studies, and wildlife species.” The document identified some instances in which sufficient evidence for endocrine-mediated effects warranted concerns, but for many hypotheses it found insufficient data to reach any definitive conclusions. The document also presented an analysis of the state of understanding of ED and prospects for future resolution of ED issues.

The 2002 WHO/IPCS document was generated over several years in a very structured manner. First, IPCS and the Organisation for Economic Co-operation and Development (OECD) convened an informal consultation in 1997; this was followed by a Scoping Meeting in 1998 to outline the objectives, scope, and development process for the document. A Steering Group of scientific experts (including two authors of this commentary, Warren Foster and Glen Van Der Kraak) was convened and met seven times over three years to provide oversight, expertise, and guidance for the project and to evaluate the accuracy, significance, and relevance of the information in the document. Several members of the Steering Group were chapter coordinators and editors that provided text; other international scientific experts served as authors for certain sections of the document. A preliminary draft of the document was circulated to several additional scientific experts and IPCS contact points for review. In total, dozens of international scientific experts contributed to the WHO/IPCS document. This report has become a guiding document in ED, as shown by more than 260 citations since its publication in 2002.

More recently, in January 2012, the European Union (EU) Directorate-General for the Environment (DG Environment) finalized and posted on the internet a separate “State of the Art Assessment of Endocrine Disrupters” (henceforth called the “SOA Assessment”), which had been commissioned in 2009 to provide a basis for developing scientific criteria for identifying endocrine disrupting chemicals (EDCs) and reviewing and possibly revising the European Community Strategy on Endocrine Disrupters^1^. The stated objectives of the study were to “(i) review the scientific knowledge published in the literature over the last 10 years and in the reports of more than 80 [EU] funded projects; (ii) review the approaches for assessment of endocrine disrupters used in selected Member States, in major competing economies outside the EU and in international bodies; and (iii) draw conclusions and answer policy relevant questions” (Kortenkamp et al., 2011). Although this report has been produced under a contract from the DG Environment, and has no connection to WHO/IPCS, it is clearly intended for the DG Environment's use as a successor to and update of the 2002 WHO/IPCS evaluation.

In the period since the WHO/IPCS review of 2002, considerable attention has focused on the potential for ED as a result of exposure to exogenous chemicals. Available information, and debate about interpretation of that information, has burgeoned. We acknowledge that it is a formidable task to review and characterize the entire state of the science - including a forthright and scientifically argued assessment of what has been established and what remains unresolved - in one document. But if this challenge is taken up - and especially if the product is presented as building on the 2002 WHO/IPCS state-of-the-science assessment - it is critical that it be done well. In our view, the SOA Assessment falls well short of what is needed, as we explain below.

Despite its title, this newer SOA Assessment did not actually conduct a full assessment of the “state of the art,” nor did it aim to do so. Rather, it constitutes a setting out of some questions with which the EU regulatory process will need to deal, mostly having to do with the plausibility that environmental chemical exposures might be able to affect endocrine-mediated processes, whether certain physiological dysfunctions might plausibly be ascribed to such effects, what the impact of simultaneous exposures to several agents might be, and whether the totality of the evidence indicates that environmentally relevant levels of exposures cause ED in humans or wildlife. While this report does address some of these questions, it takes an anecdotal approach to bringing data to bear on them; no means were developed to construct scientifically based answers to these questions in the report. To do this, one would have to (i) distinguish between apparent associations of outcomes with exposure and the inference of an ED basis for those outcomes; (ii) constitute a complete and unbiased survey of new literature since 2002; (iii) consider strengths and weaknesses and issues in interpretation of the cited literature; (iv) follow a weight-of-evidence (WOE) methodology, such as that set out in the 2002 WHO/IPCS document, for evaluation of evidence of ED; (v) document the evidence for its conclusions or the reasoning behind them; and (vi) present the evidence for or reasoning behind why conclusions that differ from those drawn in the 2002 WHO/IPCS document need to be changed.

## 2 Endocrine disruption

The SOA Assessment claims its overall objectives are to “analyse and summarise results of regulatory relevance of the scientific debate in the field of endocrine disrupting properties of substances, and to describe and characterise any relationships among the different levels of the expanded OECD conceptual framework.” In particular, the first task defined in the final report is “Analysing scientific literature on endocrine disrupters,” an exercise described as encompassing the literature with publication dates between 2000 and 2010; the results of this analysis are presented in Annex 1 of the report. Annex 1 specifies that the literature analysis is not intended to be a comprehensive scholarly review, but rather a balanced “review of reviews” that sets out to address two questions: *Can an outcome be a result of ED?* and *Can specific chemicals cause ED?* For each health outcome discussed, the SOA Assessment evaluates the evidence for an endocrine mechanism. WHO/IPCS (2002) defined an endocrine disruptor as “an exogenous substance or mixture that alters function(s) of the endocrine system and consequently causes adverse health effects in an intact organism, or its progeny, or (sub)populations.” The SOA Assessment, however, does not address the difference between endocrine *modulation* and endocrine *disruption.* Many adaptive, compensatory, and even physiologically normal and necessary processes result in measurable endocrine changes, and these cannot be considered ED. It is only when there is inappropriate expression of these natural mechanisms to such a degree that adverse effects are induced that ED occurs.

Endocrine-mediated modulation is not only normal, but essential to health. Changes considered disruptions of such mechanisms from exposure to environmental agents must be a function of the levels and timing (i.e. relative to plausible critical or vulnerable periods) of the exposure; the SOA Assessment's analyses are hampered by not evaluating these nuances. A fuller discussion of receptor-mediated biology and toxicology (e.g. dose-response, potency, receptor occupancy and affinity) is needed, as this ought to be a key aspect of an evaluation of the ability of small exposures to environmental agents to alter and disrupt normal hormonal control processes (Borgert et al., 2012). As well, the report is silent on the topic of linking changes in endocrine responses to apical outcomes. There is no discussion of normative values or intra-individual variations in hormone levels in assessing the responses to endocrine-active compounds. Such considerations are important in distinguishing the potential to interact with endocrine modulation from the circumstances that would cause dysfunction.

The SOA Assessment also does not fully consider the role of differences in endocrine signaling across animal species, and the cross-species generality of the phenomena cited is often simply presumed. Although there are similarities among humans and the various experimental animals used as models in toxicity testing, there are also important differences that must be considered when evaluating the data and determining the relevance of particular findings to human health. For example, circulating concentrations of estrogens during pregnancy are approximately 100 times or more lower in mice than in women; thus, pregnant mice may be more susceptible to the adverse effects of exposure to estrogenic compounds than are pregnant women (Witorsch, 2002). In addition, published literature available before the SOA Assessment clearly demonstrates that male rat fetuses are at least an order of magnitude more sensitive than humans to *in utero* effects of diethylstilbestrol (DES), a potent endocrine disruptor with estrogenic and anti-androgenic properties (reviewed in Borgert et al., 2012). Because endocrine modulation by exogenous chemicals must occur against the background of circulating levels of potent endogenous hormones, these examples underscore the importance of potency in determining whether and, if so, at what dose a chemical might exhibit ED properties in humans. The SOA Assessment's failure to adequately address potency issues may stem from deficiencies in its literature search and selection process (Section 3, below). To quote the candid reassessment of Richard Sharpe, who is an original proponent of the estrogen theory of testicular dysgenesis syndrome (Sharpe, 2003; not cited in the SOA Assessment):

What is reasonably clear is that all of the identified “environmental estrogens” possess weak or very weak intrinsic estrogenic activity when measured by conventional *in vitro* and *in vivo* assays for estrogenicity. By comparison with the potency of DES, for which there [are] both human and rodent data on incidence of male reproductive developmental disorders following *in utero* exposure (or neonatal exposure in rodents), it seems unlikely that any of the identified environmental compounds could induce either cryptorchidism, hypospadias or testis germ cell cancer and only a tiny possibility that such compounds could affect sperm counts/sperm production. Based on estrogenic potency, human exposure to the most potent environmental estrogens would need to be at least 1000-fold higher than this level for adverse effects relevant to the human male to be induced, and such levels of exposure are remote.

There are also differences in the ontogeny of enzyme expression, catalytic activity, and enzyme substrates across mammalian species. These differences can substantively influence test chemical effects and toxicity profiles, with implications for the relevance of experimental data to risk estimation and human health assessment. For example, rodents and humans both utilize cytochrome P450 isoenzymes in phase I detoxification, but there are differences in the isoform composition and expression of members of the CYP450 family (reviewed in Martignoni et al., 2006). Variation in CYP450 enzyme activity across species is well established, and differences could have important consequences for risk estimation and health assessment. The greatest similarities are between mice and humans, whereas rats are poor models for assessing drug and xenobiotic metabolism in humans (Turpeinen et al., 2007). Although enzyme homologues are present in different laboratory species and humans, important differences in activity may arise from differences in substrate specificity, effects of inducers and inhibitors, and mechanisms of enzyme induction (Boobis et al., 1995). For example, CYP1A1 expression and activity can be induced by environmental factors, including EDCs such as polychlorinated biphenyls (PCBs) and dioxins (e.g. 2,3,7,8 tetrachlorodibenzo-p-dioxin). The majority of polycyclic aromatic hydrocarbon toxicity is mediated through activation of the aryl hydrocarbon receptor (AhR), which leads to binding with the aryl hydrocarbon nuclear translocator (ArNT) and subsequent translocation into the nucleus. From there, the receptor ligand complex binds with the DNA in the promoter region of dioxin responsive genes, including CYP1A1. Structural differences in the AhR result in differential sensitivity of this system between species and within strains of the same species (Jana et al., 1998; Korkalainen et al., 2001, 2004). Therefore, failure to account for differences in species sensitivity and activity of CYP450 isoenzymes could critically affect risk estimation and health assessments by missing potential problems in some cases or generating overly protective safety factors in others.

Another important difference in comparative endocrinology is the role of the adrenal cortex in fetal development and parturition. The adrenal cortex plays an important role in the regulation of homeostasis during gestation and parturition in non-human primates and humans (Liggins, 1994; Ng, 2000). In humans, significant brain development occurs *in utero,* and neuroendocrine development of the hypothalamic-pituitary-adrenal axis - including development of glucocorticoid receptors - also takes place during gestation. In contrast, in mice and rats, which give birth to immature animals, much of the neuroendocrine development occurs post-natally (Matthews, 2000). Hence, important differences in the timing of brain development and the mechanisms regulating parturition across mammalian species highlights the difficulty in translating results from rodents to humans. Although comparative endocrinology can explain effects in one species but an absence of effects in humans for some outcomes, this issue is largely overlooked or ignored completely in the SOA Assessment.

Yet another critical oversight in the SOA Assessment is the importance of pharmacokinetics and the bioavailability of test agents that have been linked with potential adverse human health effects in epidemiology studies. These important issues have become a focus of recent discussions in the literature concerning the potential adverse health effects of bisphenol A (BPA). Following exposure, BPA is rapidly metabolized by phase I detoxification enzymes, resulting in only a very small fraction of BPA present in free form in the circulation (Teeguarden et al., 2011). In one study, blood and urine samples were collected hourly over 24 h from 20 volunteers who ate breakfast, lunch, and dinner at the laboratory. Their diet was enriched with canned food so they would have a high intake of BPA; average consumption of BPA was 21% higher than the 95th percentile of aggregate exposure in the adult US population. Total BPA concentrations were below the limit of detection in 86% of the blood samples tested, and free BPA was not detected in any sample studied (Teeguarden et al., 2011). Furthermore, pharmacokinetic studies in mice, rats, and rhesus monkeys suggest that adverse effects reported in rodent studies following developmental exposure would be less likely in humans based on internal dosimetry (Doerge et al., 2010, 2011; Fisher et al., 2011). Hence, failure to account for the pharmacokinetic behavior of test agents and the resulting bioavailability, together with differences in test species, could lead to predictions that exaggerate risks to human health.

When examining cases in which chemical exposures appear associated with outcomes plausibly related to ED, in practice the SOA Assessment frequently does not distinguish the questions of (i) whether some pathway other than ED could cause the outcome in principle, and (ii) whether the chemical's observed association with the outcome supports the case for ED. The SOA Assessment does not consistently consider exposure information or alternative factors that could have caused the health outcomes discussed. For example, several known risk factors (e.g. smoking, body mass index, age) are generally not given any consideration when discussing results of epidemiology studies.

The SOA Assessment does not evaluate the quality of studies (e.g. see Borgert et al., 2011) on which it bases its conclusions regarding ED. It merely states that certain studies exist, without evaluating their strengths and limitations, the use of realistic exposures, or dose-response. Most importantly, there is no evaluation of the degree of consistency among studies or the impact of significantly discordant results on the overall evaluation. While identifying the studies that address ED is an important first step in addressing the DG Environment's mandate, the evaluations in the SOA Assessment appear anecdotal rather than analytical; instead of comprehensive evaluations of the state of knowledge, its conclusions are drawn from individual endpoints without sufficient justification.

## 3 Literature search

Describing its literature search and analysis, the SOA Assessment indicates in Annex 1 that it is a “review of reviews.” To gather information on a particular topic effectively, however, one must ensure that the publications relied upon have themselves succeeded in describing and evaluating all relevant papers, the original studies reviewed received careful and critical appraisal, the included studies were interpreted correctly, and alternative interpretations were considered. These principles are consistent with what is recognized as required for conducting sound systematic reviews in other fields, notably clinical medicine (Smyth, 2000; McQueen, 2001; Gronseth, 2004; Oosterhuis et al., 2004; Weed, 2005). The SOA Assessment does not describe any process for reviewing reviews. It does not consider every review on a particular topic (nor all of the studies in each of the reviews), how the authors of each review arrive at their conclusions, or why different reviews on a controversial issue come to different conclusions.

We assume, based on the stated methodology, that the literature search aimed at capturing review articles only, although the SOA Assessment provides insufficient information (e.g. search terms, databases, subject heading descriptors, inclusion/exclusion criteria) to verify this or to allow independent replication of the search. The search appended disease terms to the primary term “endocrine disrupt*,” which ensures failure to capture reviews lacking this primary term. Because we are unable to replicate the search, and because we have not conducted our own full literature review independently, it is unclear how much literature was missed. It is clear, however, that several important reviews were omitted, among them some that took a more measured view and that bear titles using terms such as “endocrine-active” or “endocrine modulator.” To illustrate the point, the primary term “endocrine disrupt*” returns 5918 titles in PubMed, while the search “hypospadias OR cryptorchidism OR testicular dysgenesis” returns 15 639. When the two sets are combined, only 188 titles are listed (based on a literature search conducted on March 12, 2012). Neither the notable paper by Thorup et al. (2010) nor Cortes et al. (2008) is among the 188. Although the SOA Assessment cites (and discounts) Cortes et al. (2008), which reports lower incidence rates of hypospadias and cryptorchidism than some other studies, it does not cite a review by the same authors (Thorup et al., 2010) that challenges the testicular dysgenesis theory and provides alternative etiologic explanations for the observed malformations, nor does it cite other opposing analyses (Sharpe, 2003; Fisch et al., 2010). By using “endocrine disrupt*” (a term suggesting a conclusion of adverse impacts) as the primary inclusion criterion, the literature search appears to have biased the review toward studies purporting to show adverse effects of chemicals.

The SOA Assessment does not consider whether the reviews relied upon, even if published after the cutoff date of 2002, nonetheless included articles published before 2002 (and, presumably, already considered in the earlier WHO/IPCS review that it seeks to update). Further, the SOA Assessment does not estimate how many studies looked at any particular chemical and/or outcome. As discussed in more detail below, to establish the state of the science, one must consider the science as a whole; this can only be done with a comprehensive and disciplined identification of the pertinent literature.

In recent years, there has been extensive and very public scientific debate about the ED potential of several notable agents, including what endpoints might be affected and how contradictory evidence on these matters should be evaluated - a process that has included many reviews and evaluations of the evidence by expert panels convened by scientists and regulatory authorities. It would seem evident that a survey aimed at characterizing the state of the science should acknowledge these debates, present the major reviews and their findings, discuss the nature of the controversies, characterize the spectrum of opinion, and note the key new evidence that has been brought to bear on these arguments. The SOA Assessment fails to note or summarize such debates. To cite one notable example, the SOA Assessment references reviews of BPA that conclude it causes human health risks, but it fails to cite reviews that conclude that no human health risks are supportable at prevailing exposure levels (e.g. Goodman et al., 2006, 2009; Hengstler et al., 2011) and regulatory evaluations that cast doubt on the conclusions of low-exposure risk (e.g. European Commission Joint Research Centre, 2003; EFSA and ANSES, 2011). It is particularly notable that the Advisory Committee of the German Society of Toxicology, in its own in-depth evaluation of the state of the science on this particular aspect of ED, provided counterpoints to the major arguments put forth supporting adverse health effects of BPA at low exposures (Hengstler et al., 2011). Most of these counterpoints were not discussed in the SOA Assessment.

## 4 Evaluation of individual studies

The SOA Assessment does cite some individual original studies (as opposed to reviews of such studies), but it does not describe how these were selected or why other original studies were excluded. For those individual studies cited, the fundamental issue of considering the quality of the data under review (Klimisch et al., 1997) is not given systematic attention. There are generally no discussions of study methods, exposure data, statistics, biases, or issues with interpretation or generalizability. Several studies have been interpreted differently by the original authors and by different reviewers, although, in most cases, results are discussed in the SOA Assessment without a consideration of whether there is consensus regarding their interpretation or relevance to humans. For example, the SOA Assessment states that “changes in anogenital distance in humans may serve as a valuable biological marker of disruption of androgen action in foetal life,” but it does not discuss the debate regarding whether anogenital distance has any relation to ED in humans. In general, in its practice of conducting a “review of reviews,” the SOA Assessment implicitly relies on the quality evaluations of the cited reviews, but it has not examined these criteria nor their soundness and comparability across studies in the reviews relied upon.

Finally, the majority of the SOA Assessment discusses study outcomes in very general terms (e.g. association/no association, increased risk/decreased risk). Only in rare instances are actual values shown, making an assessment of the strength of association for any particular chemical and outcome impossible. Although the SOA Assessment states that it is beyond the scope of the document to assess the strength of association, one cannot determine how likely a chemical is to be causally associated with an outcome without consideration of this and several other factors, described below.

## 5 Weight-of-evidence evaluation

There are many approaches to a scientifically based WOE evaluation, but several key aspects are central (Zaza et al., 2000; Gronseth, 2004; Guzelian et al., 2005; Weed, 2005; Farquhar and Vail, 2006; Ricci et al., 2006; Goodman et al., 2006; Boobis et al., 2008; Goodman et al., 2009; Rhomberg etal., 2010; Adami etal., 2011; Borgert et al., 2011; Prueitt et al., 2011; Rhomberg etal., 2011). These include a systematic review of relevant individual studies, including an evaluation of data quality and study reliability; a systematic evaluation of consistency, specificity, and reproducibility of specific outcomes; an articulation and evaluation of hypotheses that bear on available data; and a comparison of how well each hypothesis describes the available data. The SOA Assessment was produced to review the scientific knowledge published in the literature over the last 10 years so as to inform policy decisions. Although the DG Environment mandate did not require one, we argue that a WOE evaluation is needed to inform policy decisions and provide a sound and helpful basis for addressing the challenges that the DG Environment faces in constructing its approach to the evaluation of potential EDCs. The SOA Assessment sets out to use a WOE evaluation as described in the 2002 WHO/IPCS report, but, in practice, it does not actually follow the WHO/IPCS framework nor does it formulate and follow an alternative process founded on sound WOE principles.

The framework for assessing relationships between exposures to potential EDCs and altered outcomes in the 2002 WHO/IPCS document was adapted from the Hill criteria (Hill, 1965). The framework has five main elements for evaluating scientific evidence: (i) temporality, (ii) strength of association, (iii) consistency of observations, (iv) biological plausibility, and (v) evidence of recovery following diminution of the stressor. It acknowledges scientific uncertainties, that a degree of scientific judgment is involved, and that, as they become available, additional data can change the results of assessments.

The SOA Assessment contains no discussion of temporality, and one cannot determine anything regarding the strength of association with the information provided (it generally uses subjective descriptors such as “increase” or “decrease”). There is no evaluation of whether observations are consistent within and across studies at similar exposure levels. In fact, there is no indication of how many studies were conducted for any particular chemical/outcome and how many studies were null (i.e. there is no evaluation of consistency). The SOA Assessment notes that some studies fail to find evidence of an adverse effect of a given test chemical whereas others find a positive result; it then goes on to discuss only the positive results and suggest that there is thus evidence of an endocrine mechanism and support for the supposition that EDCs are likely important causative agents. For example, the report states that the association between cryptorchidism and maternal exposure to PCBs is weak based on three studies cited that did not find an association (Hosie et al., 2000; Mol et al., 2002; McGlynn et al., 2009) and one small study that reported a positive association (Brucker-Davis et al., 2008). It seems inappropriate that one small study (56 cases and 69 controls) should form the basis for concluding that even a weak association exists when three other studies failed to find a significant association at all, especially since one of the negative studies had more statistical power (230 cases and 593 controls). Moreover, the conclusions reached are difficult to assess without information relating to the risk estimates and 95% confidence intervals reported in the studies that were reviewed.

Another factor examined in the WHO/IPCS 2002 framework but not considered in the SOA Assessment is biological plausibility. As discussed above, disruption of endocrine signaling is a question of evaluating how and by what degree exposures can perturb receptor-mediated control process, which can differ across age groups, times, and species. Doing so requires consideration of the biological basis for alteration of normal hormonal function and how this varies among species. The SOA Assessment does not assess whether effects observed in one species occur in other species or are likely to occur (or lead to adverse effects) in humans; as discussed above, the issue of chemical effects on endocrine homeostasis cannot be evaluated without consideration of comparative endocrinology.

Finally, the SOA Assessment does not evaluate alternative explanations for most outcomes. A key factor in any WOE analysis is the consideration of whether alternative hypotheses explain the data as well as - or better than - the hypothesis being tested. Despite the availability of many WOE frameworks, including the one used in the 2002 WHO/IPCS document, the SOA Assessment does not follow any framework. Instead, it selectively discusses study results, resulting in a biased evaluation that is not as useful for addressing policy questions.

## 6 Documentation of evaluation

The bases for interpretations and conclusions in the SOA Assessment are not well documented. In the conclusion of each section, the SOA Assessment lists eight criteria that are purportedly used to assess whether an adverse outcome can be attributed to an endocrine mode of action. These are general criteria that essentially determine whether there is a possible endocrine-based pathway that can lead to an effect. The SOA Assessment judges each of these as “criteria met,” “criteria mostly met,” “criteria partly met,” “evidence unclear,” “not enough data,” or “not applicable.” There is no information on how the SOA Assessment arrives at these conclusions; thus, it is lacking in transparency. The conclusions are often inconsistent with data summaries, which themselves are often biased toward reporting findings of effect over no effect. In addition, asking whether it is *possible* that an adverse outcome can be attributed to an endocrine pathway is not the germane question; rather, the relevant question is does the weight of the evidence support a causal relationship (via ED) between a specified level of exposure and an adverse health outcome? Phrasing the question as *is it possible* allows the SOA Assessment to assert “criteria met” in instances where a properly constructed WOE evaluation employing the 2002 WHO/IPCS framework would clearly indicate the totality of the evidence does not support a causal relationship.

It is notable that the criteria listed in the SOA Assessment are consistentwith the use of the recently proposed Adverse Outcome Pathway (AOP) framework, which is a conceptual framework for summarizing existing knowledge about linkages between a direct, molecular-level initiating event and an adverse outcome at a level of biological organization relevant to ecological risk assessment (Ankley et al., 2010). But the SOA Assessment criteria fail to integrate the principal element of the AOP - that perturbations of biological pathways must be sufficiently large to overcome adaptive responses before biological function is compromised. The SOA Assessment does not critically evaluate whether a realistic exposure to a named chemical has caused or could cause an adverse effect.

For example, in section 4.1.5.1, the SOA Assessment considers whether declining male reproductive health can be attributed to an endocrine disruptor. The fact that there are certain life stages that are sensitive to chemical exposure (criterion 6) does not in itself provide evidence that ED has, can, or will occur. The same argument applies in virtually all sections examined. The fact that one can draw a conceptual diagram proposing how exposure might produce an adverse outcome does not constitute proof, or even compelling evidence, that the proposed pathway is operational, much less that an adverse outcome has indeed happened or will happen. Overall, these criteria are uninformative regarding whether and under what circumstances a chemical has the potential to act as an endocrine disruptor because they are not accompanied by any critical evaluation that explains the bases for inference.

## 7 Comparison with 2002 WHO/IPCS report

Although the SOA Assessment is completely independent of the 2002 WHO/IPCS report, it clearly used the 2002 report as a baseline, aiming to extend the earlier evaluations with new information that has appeared since. The 2002 report included an evaluation of all relevant primary studies in the fields of reproductive/developmental and endocrine toxicology and underwent extensive planning and peer review. In contrast, the SOA Assessment is a self-described “review of reviews” that does not include a complete evaluation of individual studies.

Moreover, there is no discussion regarding whether the SOA Assessment comes to any interpretations or conclusions that are notably at variance with those drawn in the 2002 WHO/IPCS report. The earlier report concluded that there are some cases for which sufficient evidence for endocrine-mediated effects warranted concerns, but there were insufficient data to reach any definitive conclusions for many hypotheses. What was considered insufficient evidence in the WHO/IPCS report appears to be considered sufficient in the SOA Assessment, although one cannot be certain.

## 8 Conclusion

As we noted at the outset of these comments, considerable attention has been focused on the potential for ED by exposure to exogenous chemicals since the WHO/ IPCS review of 2002, and we applaud the resolution of the DG Environment to establish an up-to-date basis for its further policy decisions. Moving forward, sound policies must take account of this growing area of environmental science and should be based on a full understanding of all the available information, including its strengths and shortcomings, variations, inconsistencies, and outstanding questions. We recognize the challenge of accomplishing this in a single review. In our view, however, the SOA Assessment should be seen as a start that currently falls well short ofwhatwillbe needed. Itraises some issues and notes some published observations that will be relevant in addressing them, but it lacks a systematic evaluation of the literature and a rigorous basis for bringing that literature to bear on the key questions. It lacks a systematic and transparent method for selecting the studies to be included in the review, does not identify the specific literature that was reviewed, and appears to have overlooked important and significant literature critical to a balanced review. It does not note strengths and weaknesses of individual studies that ought to bear on their interpretation, and it fails to assess whether findings across studies addressing the same chemicals or endpoints find consistent results. ED is a set of modes of action, rather than a set of adverse outcome results, yet the SOA Assessment does not integrate consideration of dose-response or the underlying sciences of endocrinology and pharmacology into its evaluations. It follows no clear WOE methodology in its assessment of the interpretation of existing studies and thereby fails to support its conclusions adequately. The failure to address the evidence and reasons behind changes in conclusions *vis-a-vis* the earlier 2002 WHO/ IPCS review is especially concerning. A number of notable and highly visible scientific debates that are current in the field are not characterized or in some cases even noted, though the spectrum of opinion and the evidence adduced to support different views are undeniably a part of the “state of the science.”

In short, we maintain that a further process, utilizing a scientifically rigorous WOE evaluation focused on the specific policy issues that arise, will be necessary to form the basis for scientifically sound policy decisions as the DG Environment - and the world in general - grapples with the range of issues posed in assessing the possibility for environmental chemicals to disrupt appropriate hormonal control and function in potentially exposed organisms.

## Footnotes

1 The EU generally uses this alternate spelling of the word “disruptor.” We kept the original spelling of this and other words when referencing or quoting EU documents, policy, or programs.

## Abbreviations

AhR: aryl hydrocarbon receptor
AOP: adverse outcome pathway
ArNT: aryl hydrocarbon nuclear translocator
BPA: bishpenol A
DES: diethylstilbestrol
DG Environment: Directorate-General for the Environment
ED: endocrine disruption
EDC: endocrine-disprupting chemical
EUC: European Union
IPCS: International Programme on Chemical Safety
OECD: Organisation for Economic Co-operation and Development
PCB: polychlorinated biphenyls
WHO: World Health Organization
WOE: weight of evidence
